# Bis{[amino­(iminium­yl)meth­yl]urea} tetra­kis­{2-[(di­methyl­amino)(iminium­yl)meth­yl]guanidine} di-μ_6_-oxido-tetra-μ_3_-oxido-tetra­deca-μ_2_-oxido-octa­oxidodeca­vanadium(V) tetra­hydrate

**DOI:** 10.1107/S2414314622006277

**Published:** 2022-06-24

**Authors:** Aarón Pérez-Benítez, Jorge Luis Ariza-Ramírez, Monserrat Fortis-Valera, Rosa Elena Arroyo-Carmona, María Isabel Martínez de la Luz, Diego Ramírez-Contreras, Sylvain Bernès

**Affiliations:** a Facultad de Ciencias Químicas, Benemérita Universidad Autónoma de Puebla, 72570, Puebla, Mexico; b Instituto de Física, Benemérita Universidad Autónoma de Puebla, 72570 Puebla, Pue., Mexico; Vienna University of Technology, Austria

**Keywords:** crystal structure, vanadium, deca­vanadate, metformin, hydrogen bond

## Abstract

The title compound, (C_4_H_12_N_5_)_4_(C_2_H_7_N_4_O)_2_[V_10_O_28_]·4H_2_O, is a by-product obtained by reacting ammonium metavanadate(V), metformin hydro­chloride and acetic acid in the presence of sodium hypochlorite, at pH = 5. The crystal structure comprises a deca­vanadate(V) anion (V_10_O_28_)^6–^ lying on an inversion centre in space group *P*  , while cations and solvent water mol­ecules are placed in general positions, surrounding the anion, and forming numerous N—H⋯O and O—H⋯O hydrogen bonds. Metforminium (C_4_H_12_N_5_)^+^ and guanylurea (C_2_H_7_N_4_O)^+^ cations display the expected shape. Inter­estingly, in physiology the latter cation is known to be the main metabolite of the former one. The reported structure thus supports the role of sodium hypochlorite as an oxidizing reagent being able to degrade metformin hydro­chloride to form guanylurea.

## Abstract 2

In the crystal structure of the title compound, numerous N—H⋯O and O—H⋯O hydrogen bonds of medium strengths connect metforminium and guanylurea cations and centrosymmetric deca­vanadate(V) anions into a three-dimensional network structure.

## Graphical abstract

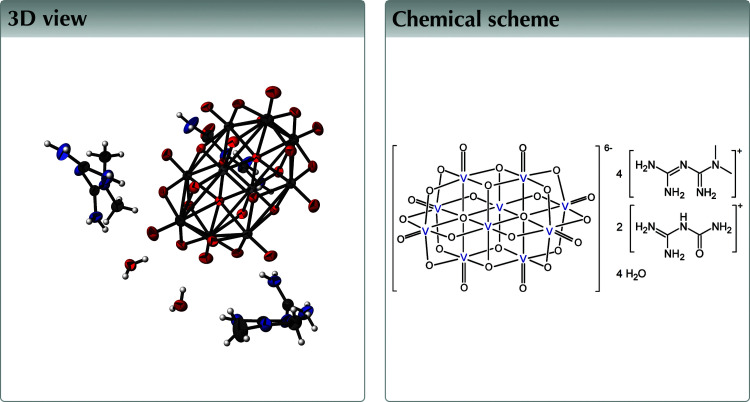

## Structure description

Metformin hydro­chloride (Metf·HCl: 1,1-di­methyl­biguanide hydro­chloride) is one of the most commonly prescribed medications for the treatment of type 2 diabetes (Maruthur *et al.*, 2016 ▸). On the other hand, coordination compounds of vanadium, including polyoxidovanadates resulting from the condensation of the vanadate anion, likewise exhibit an anti­diabetic effect, among other biological activities of inter­est in medicinal applications (Thompson *et al.*, 2009 ▸; Rehder, 2020 ▸). We are involved in studies about the chemical crystallography of compounds including both types of anti­diabetic species. In this context, we report here the crystal structure of a compound including a deca­vanadate(V) anion, metforminium cations, and a degradation product of the latter, guanylurea cation (1-carbamoylguanidinium).

**Figure 1:**
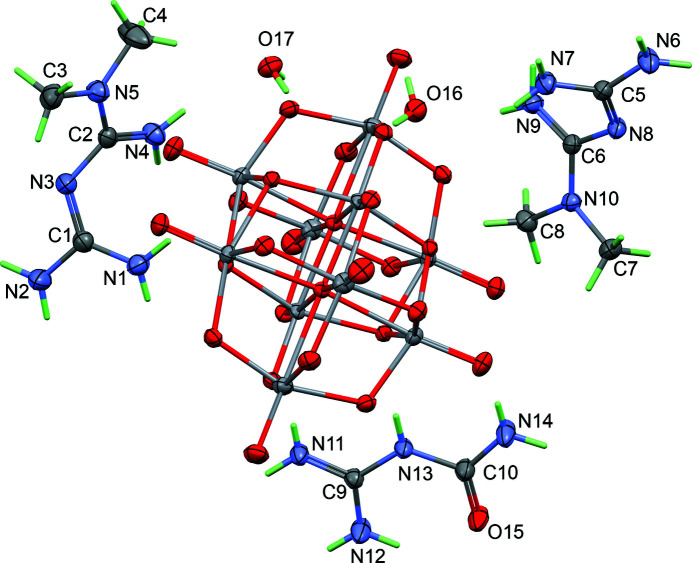
The structures of the mol­ecular entities of the title compound, with displacement ellipsoids drawn at the 40% probability level. The centrosymmetric anion is shown, while the content for cations and water mol­ecules is limited to the asymmetric unit.

The asymmetric unit of the title compound comprises one-half of the deca­vanadate(V) anion (V_10_O_28_)^6–^, three cations and two water mol­ecules of solvation. The chemical formula is thus (HMetf)_4_(HGu)_2_[V_10_O_28_]·4H_2_O, where HMetf^+^ is the metforminium cation (C_4_H_12_N_5_)^+^ and HGu^+^ is the guanylurea cation (C_2_H_7_N_4_O)^+^. All hydrogen-atom positions in the cations were obtained from difference-Fourier maps, and their positions were refined, ensuring that the right tautomers are included in the structure model (Fig. 1 ▸). The deca­vanadate(V) anion is unprotonated, and displays its usual shape, with a point-group symmetry close to *D*_2*h*_ (real *C_i_*). The twisted shape of both metforminium cations is also similar to that observed in other compounds (*e.g*. Sánchez-Lombardo *et al.*, 2014 ▸; Farzanfar *et al.*, 2015 ▸). For the first cation, the dihedral angle between C1/N1/N2/N3 and C2/C3/C4/N4/N5 mean planes is 60.39 (9)°, while the dihedral angle between the C5/N6/N7/N8 and C6/C7/C8/N9/N10 mean planes in the other metforminium cation is 58.26 (10)°. Regarding the guanylurea cation, it is nearly planar [maximum distance of 0.009 (4) Å for N12], as in a closely related salt, namely (HMetf)_2_(HGu)_4_[V_10_O_28_]·2H_2_O (Chatkon *et al.*, 2014 ▸). In the metform­inium cations, the positive charges are not clearly localized, since all C—N bond lengths span a short range, here between 1.321 (3) and 1.355 (3) Å (N—CH_3_ bonds are omitted). These cations are thus stabilized by resonance, with delocalized π-bonds, a common feature of guanidinium derivatives. In the case of the present guanylurea cation, one π-bond is probably delocalized over C9 N11 and C9 N12.

**Figure 2:**
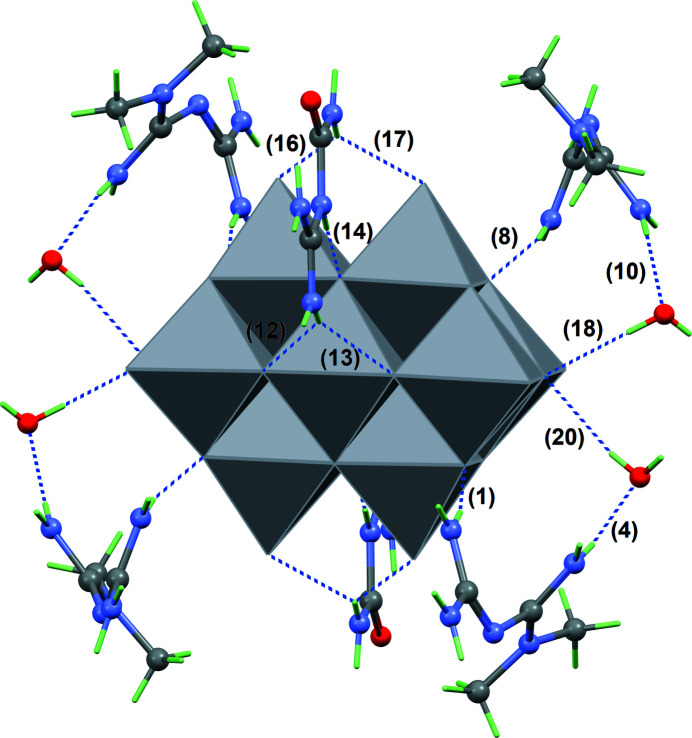
Main inter­actions between the deca­vanadate(V) anion (polyhedral representation) and the first shell including six cations and four water mol­ecules (ball-and-stick representation). Hydrogen bonds are represented by blue dashed lines, and the label associated to each hydrogen bond refers to its entry in Table 1 ▸.

**Figure 3:**
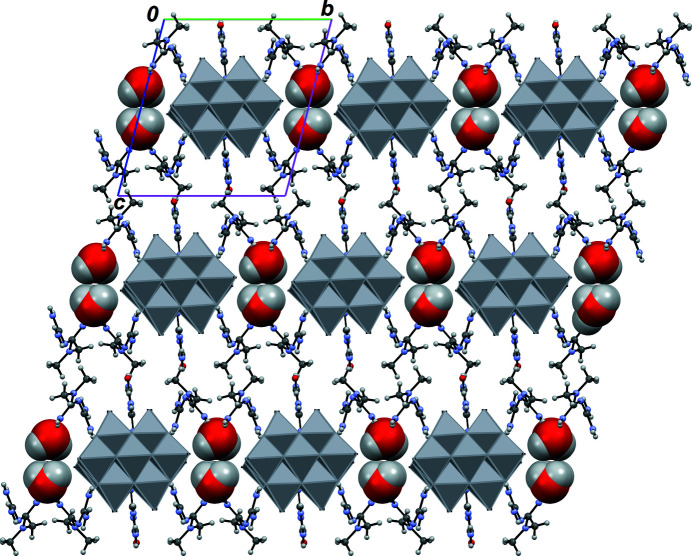
Part of the crystal structure, viewed down the *a* axis, emphasizing the positions of water mol­ecules (space-fill representation).

The cation conformations, as well as their orientations with respect to the highly charged anion favour the formation of numerous hydrogen bonds, the NH_2_ groups of HMetf^+^ and HGu^+^ being the main donors, and the O sites in the anion being the main acceptors (Table 1 ▸, Fig. 2 ▸). Empty channels oriented parallel to [100] are available in the crystal structure to accommodate water mol­ecules (O16, O17). These mol­ecules serve both as donor and acceptor groups for hydrogen bonding, and indeed form the strongest inter­molecular contacts in the crystal structure, providing cohesion between the (001) layers in which anions and cations are located (Fig. 3 ▸).

Experimental conditions used for the synthesis of the title compound were very close to those used for the synthesis of (HMetf)_2_(NH_4_)_4_[V_10_O_28_]·6H_2_O, for which we previously reported the crystal structure (Polito-Lucas *et al.*, 2021 ▸). The only difference is that sodium hypochlorite, NaOCl, was present in the reaction medium. At pH < 7, the hypochlorite anion OCl^−^ reacts with the metforminium cation, to form guanylurea (Armbruster *et al.*, 2015 ▸). Aqueous NaOCl or solid NaOCl·5H_2_O are commonly used in such oxidation processes in organic synthesis (Kirihara *et al.*, 2017 ▸). On the other hand, in physiology guanylurea is known to be the main metabolite of metformin, through a biodegradation pathway (Tassoulas *et al.*, 2021 ▸), and both mol­ecules raise a serious problem of anthropogenic contamination, since high concentrations are found in waste water (Tisler & Zwiener, 2019 ▸; Poursat *et al.*, 2019 ▸; Tucker & Wesolowski, 2020 ▸). The title compound highlights the fact that bleach, also present in waste water, has the ability to degrade metformin to guanylurea. However, the question as to whether the deca­vanadate(V) anion (or any other vanadium-containing species) promotes or inhibits metformin degradation remains open.

## Synthesis and crystallization

Orange good-quality single crystals of the title compound were obtained during the reaction between ammonium metavanadate (NH_4_VO_3_, 1.50 g, 12.1 mmol) and metformin hydro­chloride (Metf·HCl extracted from a commercial brand; 1.70 g, 10.2 mmol) in 50 ml of distilled water, 20 ml of 5% *v*/*v* acetic acid (commercial vinegar) and 2 ml of 5% *v*/*v* sodium hypochlorite (commercial bleach). In a typical procedure, NH_4_VO_3_ was dissolved by gently heating in a water bath followed by addition of Metf·HCl and stirring until dissolution. The water bath was removed, and once the mixture cooled down to room temperature, CH_3_COOH and NaOCl solutions were added. A yellow–orange homogeneous solution was obtained, and pH = 5 was measured. The solution then was evaporated at ambient conditions and the two major products, (H_2_Metf)_3_[V_10_O_28_]·8H_2_O (Sánchez-Lombardo *et al.*, 2014 ▸) and (HMetf)_4_(HGu)_2_[V_10_O_28_]·4H_2_O (estimated yields of *ca* 30 and 10%, respectively), were separated by fractional crystallization over the course of 5 to 10 d.

## Refinement

Crystal data, data collection and structure refinement details are summarized in Table 2 ▸.

## Supplementary Material

Crystal structure: contains datablock(s) I. DOI: 10.1107/S2414314622006277/wm4167sup1.cif

Structure factors: contains datablock(s) I. DOI: 10.1107/S2414314622006277/wm4167Isup2.hkl

CCDC reference: 2179058

Additional supporting information:  crystallographic information; 3D view; checkCIF report

## Figures and Tables

**Table 1:** Hydrogen-bond geometry (Å, °)

*D*—H⋯*A*	*D*—H	H⋯*A*	*D*⋯*A*	*D*—H⋯*A*
N1—H1*A*⋯O7	0.89 (3)	1.99 (3)	2.876 (3)	170 (3)
N2—H2*B*⋯O12^i^	0.85 (3)	1.99 (3)	2.835 (3)	176 (3)
N4—H4*A*⋯O16^ii^	0.87 (3)	2.03 (3)	2.893 (3)	176 (3)
N4—H4*B*⋯O17	0.79 (3)	2.13 (3)	2.908 (3)	168 (3)
N6—H6*A*⋯O15^iii^	0.91 (3)	2.08 (3)	2.984 (3)	171 (3)
N6—H6*B*⋯O9^iv^	0.80 (3)	2.15 (3)	2.947 (3)	173 (3)
N7—H7*A*⋯O10^iv^	0.75 (3)	2.64 (3)	3.362 (3)	161 (3)
N7—H7*B*⋯O13	0.81 (3)	2.23 (3)	3.031 (3)	169 (3)
N9—H9*A*⋯O17^v^	0.84 (3)	2.06 (3)	2.886 (3)	170 (3)
N9—H9*B*⋯O16	0.81 (3)	2.10 (3)	2.889 (3)	165 (3)
N11—H11*A*⋯O11^vi^	0.84 (3)	2.05 (3)	2.868 (2)	162 (3)
N11—H11*B*⋯O1	0.70 (3)	2.48 (3)	3.049 (2)	140 (3)
N11—H11*B*⋯O10	0.70 (3)	2.45 (3)	3.093 (2)	153 (3)
N13—H13⋯O6	0.82 (3)	2.11 (3)	2.926 (2)	175 (3)
N14—H14*A*⋯N8^iii^	0.88 (4)	2.22 (4)	3.091 (3)	175 (3)
N14—H14*B*⋯O2^vii^	0.74 (3)	2.44 (4)	3.055 (3)	140 (4)
N14—H14*B*⋯O4	0.74 (3)	2.53 (3)	3.156 (3)	143 (3)
O16—H16*A*⋯O8	0.80 (3)	1.97 (3)	2.770 (2)	178 (3)
O16—H16*B*⋯O3^v^	0.71 (3)	2.23 (3)	2.929 (2)	171 (4)
O17—H17*A*⋯O8	0.75 (3)	2.12 (3)	2.861 (2)	170 (3)
O17—H17*B*⋯O5^ii^	0.78 (3)	2.13 (3)	2.866 (2)	159 (3)

Symmetry codes: (i) 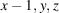 ; (ii) 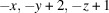 ; (iii) 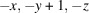 ; (iv) 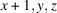 ; (v) 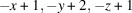 ; (vi) 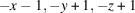 ; (vii) 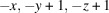 .

**Table 2:** Experimental details

Crystal data	
Chemical formula	(C_4_H_12_N_5_)_4_(C_2_H_7_N_4_O)_2_[V_10_O_28_]·4H_2_O
*M*_r_	1756.44
Crystal system, space group	Triclinic, *P* 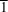
Temperature (K)	263
*a*, *b*, *c* (Å)	8.9701 (3), 13.2202 (5), 14.0861 (5)
α, β, γ (°)	99.609 (3), 103.133 (3), 107.676 (3)
*V* (Å^3^)	1499.00 (10)
*Z*	1
Radiation type	Ag *K*α, λ = 0.56083 Å
μ (mm^−1^)	0.82
Crystal size (mm)	0.35 × 0.09 × 0.08

Data collection	
Diffractometer	Stoe Stadivari
Absorption correction	Multi-scan (*X-AREA*; Stoe & Cie, 2019 ▸)
*T*_min_, *T*_max_	0.471, 1.000
No. of measured, independent and observed [*I* > 2σ(*I*)] reflections	66181, 11970, 7120
*R*_int_	0.064
(sin θ/λ)_max_ (Å^−1^)	0.782

Refinement	
*R*[*F* ^2^ > 2σ(*F* ^2^)], *wR*(*F* ^2^), *S*	0.040, 0.101, 0.83
No. of reflections	11970
No. of parameters	488
H-atom treatment	H atoms treated by a mixture of independent and constrained refinement
Δρ_max_, Δρ_min_ (e Å^−3^)	0.50, −0.75

Computer programs: *X-AREA* (Stoe & Cie, 2019 ▸), *SHELXT2018/2* (Sheldrick, 2015*a* ▸), *SHELXL2018/3* (Sheldrick, 2015*b* ▸), *Mercury* (Macrae *et al.*, 2020 ▸) and *publCIF* (Westrip, 2010 ▸).

## full crystallographic data

### Crystal data

**Table:**

(C_4_H_12_N_5_)_4_(C_2_H_7_N_4_O)_2_[V_10_O_28_]·4H_2_O	*Z* = 1
*M**_r_* = 1756.44	*F*(000) = 888
Triclinic, *P*1	*D*_x_ = 1.946 Mg m^−^^3^
*a* = 8.9701 (3) Å	Ag *K*α radiation, λ = 0.56083 Å
*b* = 13.2202 (5) Å	Cell parameters from 44097 reflections
*c* = 14.0861 (5) Å	θ = 2.2–30.9°
α = 99.609 (3)°	µ = 0.82 mm^−^^1^
β = 103.133 (3)°	*T* = 263 K
γ = 107.676 (3)°	Prism, orange
*V* = 1499.00 (10) Å^3^	0.35 × 0.09 × 0.08 mm

### Data collection

**Table:**

Stoe Stadivari diffractometer	11970 independent reflections
Radiation source: Sealed X-ray tube, Axo Astix-f Microfocus source	7120 reflections with *I* > 2σ(*I*)
Graded multilayer mirror monochromator	*R*_int_ = 0.064
Detector resolution: 5.81 pixels mm^-1^	θ_max_ = 26.0°, θ_min_ = 2.4°
ω scans	*h* = −12→14
Absorption correction: multi-scan (*X-AREA*; Stoe & Cie, 2019)	*k* = −20→20
*T*_min_ = 0.471, *T*_max_ = 1.000	*l* = −22→22
66181 measured reflections	

### Refinement

**Table:**

Refinement on *F*^2^	Primary atom site location: dual
Least-squares matrix: full	Secondary atom site location: difference Fourier map
*R*[*F*^2^ > 2σ(*F*^2^)] = 0.040	Hydrogen site location: mixed
*wR*(*F*^2^) = 0.101	H atoms treated by a mixture of independent and constrained refinement
*S* = 0.83	*w* = 1/[σ^2^(*F*_o_^2^) + (0.0523*P*)^2^] where *P* = (*F*_o_^2^ + 2*F*_c_^2^)/3
11970 reflections	(Δ/σ)_max_ = 0.001
488 parameters	Δρ_max_ = 0.50 e Å^−^^3^
0 restraints	Δρ_min_ = −0.75 e Å^−^^3^
0 constraints	

### Special details

**Table:**

Refinement. High-resolution data were collected (*d*_min_ = 0.64 Å), and all H atoms were discernible in difference-Fourier maps. Methyl H atoms were placed in calculated positions, with *U*_iso_(H) = 1.5*U*_eq_(carrier C). The positions for other H atoms were freely refined, and their isotropic displacements were calculated as *U*_iso_(H) = 1.2*U*_eq_(carrier N) and *U*_iso_(H) = 1.5*U*_eq_(carrier O).

### Fractional atomic coordinates and isotropic or equivalent isotropic displacement parameters (Å^2^)

**Table:**

	*x*	*y*	*z*	*U*_iso_*/*U*_eq_	
V1	−0.18346 (3)	0.50285 (3)	0.48387 (2)	0.02031 (7)	
V2	0.11454 (4)	0.67569 (3)	0.66199 (2)	0.02333 (7)	
V3	0.29658 (4)	0.73708 (3)	0.50272 (3)	0.02746 (8)	
V4	0.00747 (4)	0.56616 (3)	0.32784 (2)	0.02291 (7)	
V5	−0.04938 (4)	0.74348 (3)	0.47654 (3)	0.02779 (8)	
O1	−0.35393 (15)	0.39698 (12)	0.47679 (11)	0.0256 (3)	
O2	0.14821 (19)	0.71282 (13)	0.78213 (11)	0.0346 (3)	
O3	0.47419 (18)	0.82185 (14)	0.51151 (13)	0.0389 (4)	
O4	−0.02963 (18)	0.52272 (14)	0.20790 (11)	0.0334 (3)	
O5	−0.1391 (2)	0.83039 (14)	0.46158 (13)	0.0401 (4)	
O6	−0.18582 (14)	0.45457 (11)	0.34529 (9)	0.0205 (3)	
O7	−0.00402 (17)	0.75691 (12)	0.61355 (11)	0.0275 (3)	
O8	0.16426 (17)	0.82016 (12)	0.48728 (11)	0.0293 (3)	
O9	−0.08722 (16)	0.66811 (12)	0.34085 (11)	0.0266 (3)	
O10	−0.24798 (15)	0.60808 (12)	0.46689 (11)	0.0257 (3)	
O11	−0.09736 (14)	0.54661 (11)	0.62762 (10)	0.0209 (3)	
O12	0.30173 (16)	0.75443 (12)	0.64019 (11)	0.0271 (3)	
O13	0.21944 (16)	0.66484 (12)	0.36802 (11)	0.0270 (3)	
O14	0.05231 (14)	0.60398 (11)	0.49536 (10)	0.0199 (2)	
C1	−0.2523 (3)	0.8595 (2)	0.76688 (18)	0.0349 (5)	
C2	0.0296 (3)	0.95180 (18)	0.81639 (17)	0.0315 (4)	
C3	0.1549 (3)	0.8988 (2)	0.96161 (19)	0.0466 (6)	
H3A	0.191943	0.952085	1.025219	0.070*	
H3B	0.222871	0.854931	0.962427	0.070*	
H3C	0.043527	0.852377	0.950131	0.070*	
C4	0.3258 (3)	1.0073 (4)	0.8715 (3)	0.0729 (11)	
H4C	0.344844	0.959712	0.819942	0.109*	
H4D	0.407592	1.021958	0.934421	0.109*	
H4E	0.331692	1.075066	0.853596	0.109*	
N1	−0.2665 (3)	0.79810 (18)	0.67858 (16)	0.0376 (5)	
H1A	−0.178 (3)	0.794 (2)	0.662 (2)	0.045*	
H1B	−0.351 (3)	0.771 (2)	0.636 (2)	0.045*	
N2	−0.3897 (3)	0.8602 (2)	0.7888 (2)	0.0537 (7)	
H2A	−0.379 (4)	0.902 (3)	0.848 (3)	0.064*	
H2B	−0.484 (4)	0.829 (3)	0.747 (3)	0.064*	
N3	−0.1127 (2)	0.91530 (19)	0.83847 (15)	0.0404 (5)	
N4	0.0405 (3)	0.99452 (18)	0.73832 (17)	0.0370 (4)	
H4A	−0.045 (3)	1.001 (2)	0.700 (2)	0.044*	
H4B	0.117 (3)	1.006 (2)	0.717 (2)	0.044*	
N5	0.1645 (2)	0.95465 (18)	0.88149 (16)	0.0380 (4)	
C5	0.5314 (2)	0.70581 (19)	0.20996 (16)	0.0304 (4)	
C6	0.3190 (2)	0.77189 (18)	0.17505 (15)	0.0271 (4)	
C7	0.0868 (3)	0.6332 (2)	0.04521 (17)	0.0366 (5)	
H7C	0.083349	0.648205	−0.019487	0.055*	
H7D	−0.022500	0.596148	0.046520	0.055*	
H7E	0.150763	0.587596	0.057091	0.055*	
C8	0.0538 (3)	0.7913 (2)	0.15167 (19)	0.0371 (5)	
H8A	0.034360	0.776677	0.213139	0.056*	
H8B	−0.048504	0.764710	0.099138	0.056*	
H8C	0.104801	0.868982	0.161395	0.056*	
N6	0.6537 (2)	0.6869 (2)	0.18114 (18)	0.0435 (5)	
H6A	0.660 (3)	0.685 (2)	0.117 (2)	0.052*	
H6B	0.720 (4)	0.676 (3)	0.222 (2)	0.052*	
N7	0.5283 (3)	0.70352 (19)	0.30382 (16)	0.0378 (5)	
H7A	0.595 (3)	0.695 (2)	0.341 (2)	0.045*	
H7B	0.449 (3)	0.702 (2)	0.323 (2)	0.045*	
N8	0.4152 (2)	0.72119 (17)	0.14230 (13)	0.0314 (4)	
N9	0.3795 (2)	0.86178 (18)	0.25014 (16)	0.0355 (4)	
H9A	0.479 (3)	0.886 (2)	0.283 (2)	0.043*	
H9B	0.327 (3)	0.891 (2)	0.277 (2)	0.043*	
N10	0.16091 (19)	0.73611 (15)	0.12336 (13)	0.0285 (4)	
C9	−0.6276 (2)	0.42463 (19)	0.23836 (16)	0.0289 (4)	
C10	−0.5215 (3)	0.3700 (2)	0.10014 (17)	0.0366 (5)	
N11	−0.5989 (2)	0.4617 (2)	0.33434 (15)	0.0375 (5)	
H11A	−0.674 (3)	0.468 (2)	0.358 (2)	0.045*	
H11B	−0.522 (3)	0.477 (2)	0.370 (2)	0.045*	
N12	−0.7731 (3)	0.4028 (3)	0.17683 (18)	0.0548 (7)	
H12A	−0.785 (4)	0.375 (3)	0.117 (3)	0.066*	
H12B	−0.846 (4)	0.412 (3)	0.200 (3)	0.066*	
N13	−0.5049 (2)	0.41034 (18)	0.20225 (14)	0.0327 (4)	
H13	−0.418 (3)	0.424 (2)	0.245 (2)	0.039*	
N14	−0.3872 (3)	0.3626 (3)	0.08178 (18)	0.0559 (7)	
H14A	−0.396 (4)	0.343 (3)	0.018 (3)	0.067*	
H14B	−0.309 (4)	0.376 (3)	0.122 (3)	0.067*	
O15	−0.6510 (2)	0.3447 (2)	0.03409 (13)	0.0559 (6)	
O16	0.2449 (2)	0.97674 (15)	0.38087 (13)	0.0352 (4)	
H16A	0.220 (4)	0.931 (3)	0.411 (2)	0.053*	
H16B	0.312 (4)	1.023 (3)	0.412 (2)	0.053*	
O17	0.2829 (2)	1.02959 (15)	0.62909 (14)	0.0364 (4)	
H17A	0.247 (4)	0.972 (3)	0.597 (2)	0.055*	
H17B	0.243 (4)	1.057 (3)	0.591 (2)	0.055*	

### Atomic displacement parameters (Å^2^)

**Table:**

	*U*^11^	*U*^22^	*U*^33^	*U*^12^	*U*^13^	*U*^23^
V1	0.01787 (12)	0.02549 (16)	0.01941 (15)	0.01000 (12)	0.00608 (11)	0.00545 (12)
V2	0.02600 (14)	0.02310 (17)	0.01967 (16)	0.00949 (13)	0.00553 (12)	0.00253 (13)
V3	0.02363 (14)	0.02576 (18)	0.02969 (18)	0.00416 (13)	0.00690 (13)	0.00857 (14)
V4	0.02465 (14)	0.02587 (17)	0.01891 (15)	0.00888 (13)	0.00673 (12)	0.00732 (13)
V5	0.03017 (16)	0.02440 (17)	0.03055 (19)	0.01368 (14)	0.00625 (14)	0.00780 (14)
O1	0.0188 (5)	0.0315 (8)	0.0268 (7)	0.0086 (5)	0.0069 (5)	0.0084 (6)
O2	0.0442 (8)	0.0349 (9)	0.0224 (7)	0.0163 (7)	0.0071 (6)	0.0013 (6)
O3	0.0305 (7)	0.0358 (9)	0.0407 (9)	−0.0010 (7)	0.0081 (7)	0.0123 (7)
O4	0.0363 (7)	0.0405 (9)	0.0220 (7)	0.0115 (7)	0.0086 (6)	0.0084 (7)
O5	0.0462 (9)	0.0349 (9)	0.0428 (10)	0.0235 (8)	0.0069 (8)	0.0104 (8)
O6	0.0198 (5)	0.0232 (7)	0.0181 (6)	0.0078 (5)	0.0047 (5)	0.0049 (5)
O7	0.0328 (7)	0.0256 (7)	0.0254 (7)	0.0154 (6)	0.0061 (6)	0.0037 (6)
O8	0.0324 (7)	0.0210 (7)	0.0319 (8)	0.0073 (6)	0.0069 (6)	0.0084 (6)
O9	0.0290 (6)	0.0272 (7)	0.0251 (7)	0.0125 (6)	0.0051 (5)	0.0096 (6)
O10	0.0227 (6)	0.0292 (7)	0.0275 (7)	0.0138 (5)	0.0059 (5)	0.0065 (6)
O11	0.0208 (5)	0.0257 (7)	0.0184 (6)	0.0105 (5)	0.0075 (5)	0.0046 (5)
O12	0.0257 (6)	0.0245 (7)	0.0246 (7)	0.0041 (5)	0.0042 (5)	0.0030 (6)
O13	0.0246 (6)	0.0304 (8)	0.0261 (7)	0.0066 (6)	0.0095 (5)	0.0103 (6)
O14	0.0194 (5)	0.0210 (7)	0.0200 (6)	0.0084 (5)	0.0055 (5)	0.0055 (5)
C1	0.0335 (10)	0.0367 (12)	0.0359 (12)	0.0124 (9)	0.0124 (9)	0.0098 (10)
C2	0.0325 (9)	0.0275 (11)	0.0333 (11)	0.0109 (8)	0.0118 (9)	0.0012 (9)
C3	0.0523 (14)	0.0520 (16)	0.0332 (13)	0.0174 (13)	0.0097 (11)	0.0111 (12)
C4	0.0323 (12)	0.113 (3)	0.076 (2)	0.0184 (16)	0.0120 (14)	0.051 (2)
N1	0.0327 (9)	0.0389 (12)	0.0353 (11)	0.0124 (9)	0.0053 (8)	0.0012 (9)
N2	0.0298 (9)	0.0704 (18)	0.0462 (14)	0.0106 (11)	0.0101 (9)	−0.0085 (12)
N3	0.0301 (8)	0.0516 (13)	0.0327 (10)	0.0090 (9)	0.0103 (8)	0.0020 (9)
N4	0.0321 (9)	0.0411 (12)	0.0423 (12)	0.0162 (9)	0.0128 (9)	0.0141 (9)
N5	0.0317 (8)	0.0433 (12)	0.0386 (11)	0.0123 (8)	0.0100 (8)	0.0120 (9)
C5	0.0282 (9)	0.0331 (11)	0.0271 (10)	0.0095 (8)	0.0077 (8)	0.0038 (9)
C6	0.0273 (8)	0.0294 (10)	0.0243 (9)	0.0092 (8)	0.0083 (7)	0.0070 (8)
C7	0.0336 (10)	0.0374 (13)	0.0309 (12)	0.0082 (9)	0.0052 (9)	0.0025 (10)
C8	0.0314 (10)	0.0420 (14)	0.0408 (13)	0.0174 (10)	0.0118 (9)	0.0080 (11)
N6	0.0342 (9)	0.0738 (17)	0.0334 (11)	0.0310 (11)	0.0113 (8)	0.0181 (11)
N7	0.0344 (9)	0.0522 (13)	0.0315 (11)	0.0191 (9)	0.0107 (8)	0.0144 (9)
N8	0.0292 (8)	0.0433 (11)	0.0236 (8)	0.0179 (8)	0.0069 (7)	0.0051 (8)
N9	0.0273 (8)	0.0364 (11)	0.0363 (11)	0.0098 (8)	0.0077 (8)	−0.0022 (8)
N10	0.0250 (7)	0.0304 (9)	0.0276 (9)	0.0095 (7)	0.0060 (6)	0.0042 (7)
C9	0.0263 (8)	0.0365 (12)	0.0267 (10)	0.0142 (8)	0.0086 (8)	0.0084 (9)
C10	0.0320 (10)	0.0531 (15)	0.0264 (11)	0.0190 (10)	0.0085 (8)	0.0072 (10)
N11	0.0277 (8)	0.0597 (14)	0.0259 (10)	0.0196 (9)	0.0083 (7)	0.0051 (9)
N12	0.0317 (9)	0.103 (2)	0.0298 (11)	0.0350 (12)	0.0054 (9)	0.0002 (12)
N13	0.0239 (7)	0.0504 (12)	0.0242 (9)	0.0169 (8)	0.0060 (7)	0.0052 (8)
N14	0.0376 (10)	0.107 (2)	0.0276 (11)	0.0385 (13)	0.0090 (9)	0.0048 (13)
O15	0.0343 (8)	0.1055 (18)	0.0247 (8)	0.0323 (10)	0.0029 (7)	0.0022 (10)
O16	0.0415 (9)	0.0307 (9)	0.0326 (9)	0.0109 (7)	0.0097 (7)	0.0115 (7)
O17	0.0372 (8)	0.0302 (9)	0.0353 (9)	0.0093 (7)	0.0043 (7)	0.0050 (7)

### Geometric parameters (Å, º)

**Table:**

V1—O10	1.6925 (14)	C4—N5	1.454 (3)
V1—O1	1.7005 (13)	C4—H4C	0.9600
V1—O11	1.9126 (13)	C4—H4D	0.9600
V1—O6	1.9430 (13)	C4—H4E	0.9600
V1—O14	2.0836 (12)	N1—H1A	0.89 (3)
V1—O14^i^	2.1105 (13)	N1—H1B	0.79 (3)
V1—V5	3.0691 (5)	N2—H2A	0.89 (4)
V1—V3^i^	3.0755 (5)	N2—H2B	0.85 (3)
V2—O2	1.6113 (15)	N4—H4A	0.87 (3)
V2—O12	1.8102 (14)	N4—H4B	0.79 (3)
V2—O7	1.8324 (14)	C5—N6	1.329 (3)
V2—O6^i^	2.0062 (14)	C5—N8	1.334 (3)
V2—O11	2.0238 (13)	C5—N7	1.334 (3)
V2—O14	2.2495 (13)	C6—N9	1.324 (3)
V2—V4^i^	3.0888 (5)	C6—N10	1.331 (2)
V2—V5	3.1024 (5)	C6—N8	1.355 (3)
V3—O3	1.6091 (15)	C7—N10	1.457 (3)
V3—O13	1.8407 (15)	C7—H7C	0.9600
V3—O8	1.8484 (15)	C7—H7D	0.9600
V3—O12	1.8998 (15)	C7—H7E	0.9600
V3—O1^i^	2.0361 (15)	C8—N10	1.455 (3)
V3—O14	2.3243 (12)	C8—H8A	0.9600
V3—V5	3.0697 (5)	C8—H8B	0.9600
V3—V4	3.0951 (5)	C8—H8C	0.9600
V4—O4	1.6122 (15)	N6—H6A	0.91 (3)
V4—O9	1.8042 (15)	N6—H6B	0.80 (3)
V4—O13	1.8427 (13)	N7—H7A	0.75 (3)
V4—O6	2.0012 (13)	N7—H7B	0.81 (3)
V4—O11^i^	2.0196 (14)	N9—H9A	0.84 (3)
V4—O14	2.2434 (13)	N9—H9B	0.81 (3)
V4—V5	3.1169 (5)	C9—N11	1.296 (3)
V5—O5	1.6054 (16)	C9—N12	1.309 (3)
V5—O8	1.8331 (14)	C9—N13	1.361 (3)
V5—O7	1.8457 (15)	C10—O15	1.223 (3)
V5—O9	1.9040 (15)	C10—N14	1.316 (3)
V5—O10	2.0660 (14)	C10—N13	1.405 (3)
V5—O14	2.3191 (13)	N11—H11A	0.84 (3)
C1—N3	1.321 (3)	N11—H11B	0.70 (3)
C1—N1	1.323 (3)	N12—H12A	0.83 (3)
C1—N2	1.339 (3)	N12—H12B	0.82 (3)
C2—N4	1.327 (3)	N13—H13	0.82 (3)
C2—N5	1.328 (3)	N14—H14A	0.88 (4)
C2—N3	1.347 (3)	N14—H14B	0.74 (3)
C3—N5	1.453 (3)	O16—H16A	0.80 (3)
C3—H3A	0.9600	O16—H16B	0.71 (3)
C3—H3B	0.9600	O17—H17A	0.75 (3)
C3—H3C	0.9600	O17—H17B	0.78 (3)
			
O10—V1—O1	105.46 (7)	O10—V5—O14	74.12 (5)
O10—V1—O11	98.71 (6)	O5—V5—V1	131.07 (7)
O1—V1—O11	96.92 (6)	O8—V5—V1	125.08 (5)
O10—V1—O6	95.97 (6)	O7—V5—V1	78.36 (5)
O1—V1—O6	95.74 (6)	O9—V5—V1	78.38 (4)
O11—V1—O6	157.30 (5)	O10—V5—V1	31.41 (4)
O10—V1—O14	88.53 (6)	O14—V5—V1	42.73 (3)
O1—V1—O14	165.98 (6)	O5—V5—V3	137.49 (7)
O11—V1—O14	81.62 (5)	O8—V5—V3	33.66 (5)
O6—V1—O14	81.53 (5)	O7—V5—V3	85.71 (4)
O10—V1—O14^i^	167.14 (6)	O9—V5—V3	83.45 (4)
O1—V1—O14^i^	87.35 (6)	O10—V5—V3	122.77 (4)
O11—V1—O14^i^	80.52 (5)	O14—V5—V3	48.69 (3)
O6—V1—O14^i^	81.33 (5)	V1—V5—V3	91.420 (13)
O14—V1—O14^i^	78.65 (5)	O5—V5—V2	134.32 (7)
O10—V1—V5	39.51 (5)	O8—V5—V2	82.26 (5)
O1—V1—V5	144.96 (5)	O7—V5—V2	32.36 (4)
O11—V1—V5	89.98 (4)	O9—V5—V2	123.48 (4)
O6—V1—V5	90.41 (4)	O10—V5—V2	82.77 (4)
O14—V1—V5	49.05 (4)	O14—V5—V2	46.29 (3)
O14^i^—V1—V5	127.69 (3)	V1—V5—V2	61.613 (12)
O10—V1—V3^i^	143.79 (5)	V3—V5—V2	61.042 (11)
O1—V1—V3^i^	38.33 (5)	O5—V5—V4	133.62 (7)
O11—V1—V3^i^	88.31 (4)	O8—V5—V4	81.96 (5)
O6—V1—V3^i^	90.02 (4)	O7—V5—V4	123.82 (5)
O14—V1—V3^i^	127.68 (4)	O9—V5—V4	31.82 (4)
O14^i^—V1—V3^i^	49.04 (3)	O10—V5—V4	80.43 (4)
V5—V1—V3^i^	176.557 (14)	O14—V5—V4	45.90 (3)
O2—V2—O12	104.71 (7)	V1—V5—V4	61.206 (11)
O2—V2—O7	103.32 (7)	V3—V5—V4	60.033 (12)
O12—V2—O7	95.37 (7)	V2—V5—V4	91.958 (13)
O2—V2—O6^i^	99.03 (7)	V1—O1—V3^i^	110.47 (6)
O12—V2—O6^i^	89.97 (6)	V1—O6—V4	105.96 (6)
O7—V2—O6^i^	154.83 (6)	V1—O6—V2^i^	106.54 (6)
O2—V2—O11	98.92 (7)	V4—O6—V2^i^	100.85 (6)
O12—V2—O11	154.25 (6)	V2—O7—V5	115.02 (8)
O7—V2—O11	88.94 (6)	V5—O8—V3	112.99 (8)
O6^i^—V2—O11	76.20 (5)	V4—O9—V5	114.37 (7)
O2—V2—O14	173.33 (7)	V1—O10—V5	109.08 (6)
O12—V2—O14	80.50 (6)	V1—O11—V4^i^	108.12 (6)
O7—V2—O14	80.05 (6)	V1—O11—V2	106.80 (6)
O6^i^—V2—O14	76.60 (5)	V4^i^—O11—V2	99.62 (5)
O11—V2—O14	75.26 (5)	V2—O12—V3	115.31 (7)
O2—V2—V4^i^	88.84 (6)	V3—O13—V4	114.34 (7)
O12—V2—V4^i^	129.46 (5)	V1—O14—V1^i^	101.35 (5)
O7—V2—V4^i^	129.07 (5)	V1—O14—V4	93.34 (5)
O6^i^—V2—V4^i^	39.52 (4)	V1^i^—O14—V4	93.95 (5)
O11—V2—V4^i^	40.14 (4)	V1—O14—V2	93.60 (5)
O14—V2—V4^i^	84.60 (3)	V1^i^—O14—V2	93.05 (5)
O2—V2—V5	135.78 (6)	V4—O14—V2	168.99 (7)
O12—V2—V5	83.17 (5)	V1—O14—V5	88.22 (4)
O7—V2—V5	32.62 (5)	V1^i^—O14—V5	170.40 (6)
O6^i^—V2—V5	124.76 (4)	V4—O14—V5	86.16 (4)
O11—V2—V5	87.04 (4)	V2—O14—V5	85.53 (5)
O14—V2—V5	48.18 (3)	V1—O14—V3	170.95 (7)
V4^i^—V2—V5	119.903 (14)	V1^i^—O14—V3	87.68 (4)
O3—V3—O13	102.26 (8)	V4—O14—V3	85.29 (4)
O3—V3—O8	103.30 (8)	V2—O14—V3	86.51 (4)
O13—V3—O8	92.71 (7)	V5—O14—V3	82.77 (4)
O3—V3—O12	101.81 (7)	N3—C1—N1	125.0 (2)
O13—V3—O12	154.56 (6)	N3—C1—N2	116.8 (2)
O8—V3—O12	89.66 (7)	N1—C1—N2	118.1 (2)
O3—V3—O1^i^	100.28 (7)	N4—C2—N5	119.8 (2)
O13—V3—O1^i^	85.14 (6)	N4—C2—N3	122.1 (2)
O8—V3—O1^i^	156.22 (6)	N5—C2—N3	117.8 (2)
O12—V3—O1^i^	82.61 (6)	N5—C3—H3A	109.5
O3—V3—O14	174.67 (7)	N5—C3—H3B	109.5
O13—V3—O14	78.49 (5)	H3A—C3—H3B	109.5
O8—V3—O14	81.89 (5)	N5—C3—H3C	109.5
O12—V3—O14	76.79 (5)	H3A—C3—H3C	109.5
O1^i^—V3—O14	74.47 (5)	H3B—C3—H3C	109.5
O3—V3—V5	136.65 (7)	N5—C4—H4C	109.5
O13—V3—V5	85.37 (4)	N5—C4—H4D	109.5
O8—V3—V5	33.35 (4)	H4C—C4—H4D	109.5
O12—V3—V5	82.78 (4)	N5—C4—H4E	109.5
O1^i^—V3—V5	122.98 (4)	H4C—C4—H4E	109.5
O14—V3—V5	48.54 (3)	H4D—C4—H4E	109.5
O3—V3—V1^i^	131.48 (7)	C1—N1—H1A	120.9 (18)
O13—V3—V1^i^	79.52 (5)	C1—N1—H1B	121 (2)
O8—V3—V1^i^	125.17 (5)	H1A—N1—H1B	117 (3)
O12—V3—V1^i^	78.46 (5)	C1—N2—H2A	117 (2)
O1^i^—V3—V1^i^	31.20 (4)	C1—N2—H2B	123 (2)
O14—V3—V1^i^	43.29 (3)	H2A—N2—H2B	119 (3)
V5—V3—V1^i^	91.829 (13)	C1—N3—C2	121.3 (2)
O3—V3—V4	134.99 (6)	C2—N4—H4A	120.2 (18)
O13—V3—V4	32.85 (4)	C2—N4—H4B	125 (2)
O8—V3—V4	82.37 (5)	H4A—N4—H4B	114 (3)
O12—V3—V4	123.02 (4)	C2—N5—C3	120.9 (2)
O1^i^—V3—V4	83.12 (4)	C2—N5—C4	121.5 (2)
O14—V3—V4	46.25 (3)	C3—N5—C4	117.5 (2)
V5—V3—V4	60.738 (12)	N6—C5—N8	118.3 (2)
V1^i^—V3—V4	62.131 (11)	N6—C5—N7	117.9 (2)
O4—V4—O9	104.49 (7)	N8—C5—N7	123.7 (2)
O4—V4—O13	103.24 (7)	N9—C6—N10	119.0 (2)
O9—V4—O13	95.73 (7)	N9—C6—N8	122.24 (18)
O4—V4—O6	98.59 (7)	N10—C6—N8	118.44 (19)
O9—V4—O6	90.50 (6)	N10—C7—H7C	109.5
O13—V4—O6	154.96 (6)	N10—C7—H7D	109.5
O4—V4—O11^i^	98.58 (7)	H7C—C7—H7D	109.5
O9—V4—O11^i^	154.97 (6)	N10—C7—H7E	109.5
O13—V4—O11^i^	88.24 (6)	H7C—C7—H7E	109.5
O6—V4—O11^i^	76.41 (5)	H7D—C7—H7E	109.5
O4—V4—O14	172.62 (7)	N10—C8—H8A	109.5
O9—V4—O14	81.17 (6)	N10—C8—H8B	109.5
O13—V4—O14	80.64 (5)	H8A—C8—H8B	109.5
O6—V4—O14	76.39 (5)	N10—C8—H8C	109.5
O11^i^—V4—O14	75.10 (5)	H8A—C8—H8C	109.5
O4—V4—V2^i^	88.30 (6)	H8B—C8—H8C	109.5
O9—V4—V2^i^	130.12 (5)	C5—N6—H6A	121.6 (18)
O13—V4—V2^i^	128.48 (5)	C5—N6—H6B	116 (2)
O6—V4—V2^i^	39.63 (4)	H6A—N6—H6B	122 (3)
O11^i^—V4—V2^i^	40.24 (4)	C5—N7—H7A	122 (2)
O14—V4—V2^i^	84.39 (3)	C5—N7—H7B	123 (2)
O4—V4—V3	135.96 (6)	H7A—N7—H7B	115 (3)
O9—V4—V3	84.25 (5)	C5—N8—C6	118.98 (18)
O13—V4—V3	32.81 (5)	C6—N9—H9A	120 (2)
O6—V4—V3	124.78 (4)	C6—N9—H9B	126 (2)
O11^i^—V4—V3	85.92 (4)	H9A—N9—H9B	112 (3)
O14—V4—V3	48.45 (3)	C6—N10—C8	120.82 (19)
V2^i^—V4—V3	119.121 (14)	C6—N10—C7	120.68 (19)
O4—V4—V5	138.15 (6)	C8—N10—C7	118.08 (17)
O9—V4—V5	33.81 (5)	N11—C9—N12	119.9 (2)
O13—V4—V5	83.95 (5)	N11—C9—N13	119.58 (19)
O6—V4—V5	87.98 (4)	N12—C9—N13	120.6 (2)
O11^i^—V4—V5	123.03 (4)	O15—C10—N14	123.2 (2)
O14—V4—V5	47.94 (3)	O15—C10—N13	122.1 (2)
V2^i^—V4—V5	119.587 (14)	N14—C10—N13	114.7 (2)
V3—V4—V5	59.228 (12)	C9—N11—H11A	121.0 (19)
O5—V5—O8	103.84 (8)	C9—N11—H11B	124 (2)
O5—V5—O7	102.08 (8)	H11A—N11—H11B	116 (3)
O8—V5—O7	92.75 (7)	C9—N12—H12A	115 (2)
O5—V5—O9	101.85 (8)	C9—N12—H12B	119 (2)
O8—V5—O9	91.20 (7)	H12A—N12—H12B	126 (3)
O7—V5—O9	154.03 (7)	C9—N13—C10	124.73 (18)
O5—V5—O10	99.67 (7)	C9—N13—H13	115.2 (19)
O8—V5—O10	156.41 (6)	C10—N13—H13	120.0 (19)
O7—V5—O10	84.18 (6)	C10—N14—H14A	115 (2)
O9—V5—O10	82.01 (6)	C10—N14—H14B	123 (3)
O5—V5—O14	173.78 (7)	H14A—N14—H14B	122 (3)
O8—V5—O14	82.35 (5)	H16A—O16—H16B	111 (3)
O7—V5—O14	77.92 (5)	H17A—O17—H17B	99 (3)
O9—V5—O14	77.19 (5)		
			
O10—V1—O1—V3^i^	−179.43 (7)	O7—V2—O12—V3	−70.42 (9)
O11—V1—O1—V3^i^	−78.38 (8)	O6^i^—V2—O12—V3	84.95 (8)
O6—V1—O1—V3^i^	82.74 (7)	O11—V2—O12—V3	28.32 (19)
O14—V1—O1—V3^i^	4.8 (3)	O14—V2—O12—V3	8.53 (7)
O14^i^—V1—O1—V3^i^	1.73 (7)	V4^i^—V2—O12—V3	83.39 (9)
V5—V1—O1—V3^i^	−178.31 (3)	V5—V2—O12—V3	−40.10 (7)
O2—V2—O7—V5	174.91 (8)	O3—V3—O12—V2	176.91 (9)
O12—V2—O7—V5	68.39 (9)	O13—V3—O12—V2	−22.2 (2)
O6^i^—V2—O7—V5	−33.07 (19)	O8—V3—O12—V2	73.38 (9)
O11—V2—O7—V5	−86.17 (8)	O1^i^—V3—O12—V2	−84.07 (8)
O14—V2—O7—V5	−10.96 (7)	O14—V3—O12—V2	−8.37 (7)
V4^i^—V2—O7—V5	−85.57 (8)	V5—V3—O12—V2	40.66 (7)
O5—V5—O7—V2	−175.65 (9)	V1^i^—V3—O12—V2	−52.71 (7)
O8—V5—O7—V2	−70.87 (9)	V4—V3—O12—V2	−7.28 (11)
O9—V5—O7—V2	27.55 (19)	O3—V3—O13—V4	−175.67 (9)
O10—V5—O7—V2	85.67 (8)	O8—V3—O13—V4	−71.43 (9)
O14—V5—O7—V2	10.70 (7)	O12—V3—O13—V4	23.5 (2)
V1—V5—O7—V2	54.41 (7)	O1^i^—V3—O13—V4	84.84 (8)
V3—V5—O7—V2	−37.93 (7)	O14—V3—O13—V4	9.73 (7)
V4—V5—O7—V2	11.29 (10)	V5—V3—O13—V4	−38.87 (7)
O5—V5—O8—V3	−178.80 (9)	V1^i^—V3—O13—V4	53.86 (7)
O7—V5—O8—V3	78.04 (9)	O4—V4—O13—V3	176.40 (9)
O9—V5—O8—V3	−76.28 (8)	O9—V4—O13—V3	70.03 (9)
O10—V5—O8—V3	−3.7 (2)	O6—V4—O13—V3	−33.6 (2)
O14—V5—O8—V3	0.62 (7)	O11^i^—V4—O13—V3	−85.20 (8)
V1—V5—O8—V3	0.33 (11)	O14—V4—O13—V3	−10.01 (7)
V2—V5—O8—V3	47.35 (7)	V2^i^—V4—O13—V3	−85.08 (9)
V4—V5—O8—V3	−45.74 (7)	V5—V4—O13—V3	38.28 (7)
O3—V3—O8—V5	−179.37 (9)	N1—C1—N3—C2	27.6 (4)
O13—V3—O8—V5	77.35 (8)	N2—C1—N3—C2	−156.9 (3)
O12—V3—O8—V5	−77.32 (8)	N4—C2—N3—C1	40.9 (4)
O1^i^—V3—O8—V5	−6.7 (2)	N5—C2—N3—C1	−146.1 (2)
O14—V3—O8—V5	−0.62 (7)	N4—C2—N5—C3	−173.7 (2)
V1^i^—V3—O8—V5	−1.73 (11)	N3—C2—N5—C3	13.1 (4)
V4—V3—O8—V5	46.10 (7)	N4—C2—N5—C4	2.3 (4)
O4—V4—O9—V5	−175.39 (8)	N3—C2—N5—C4	−170.9 (3)
O13—V4—O9—V5	−70.10 (8)	N6—C5—N8—C6	−160.5 (2)
O6—V4—O9—V5	85.61 (8)	N7—C5—N8—C6	22.6 (3)
O11^i^—V4—O9—V5	28.06 (18)	N9—C6—N8—C5	44.9 (3)
O14—V4—O9—V5	9.47 (7)	N10—C6—N8—C5	−141.8 (2)
V2^i^—V4—O9—V5	84.38 (8)	N9—C6—N10—C8	−3.0 (3)
V3—V4—O9—V5	−39.32 (6)	N8—C6—N10—C8	−176.5 (2)
O1—V1—O10—V5	178.99 (7)	N9—C6—N10—C7	−175.4 (2)
O11—V1—O10—V5	79.26 (7)	N8—C6—N10—C7	11.1 (3)
O6—V1—O10—V5	−83.36 (7)	N11—C9—N13—C10	179.5 (2)
O14—V1—O10—V5	−2.03 (7)	N12—C9—N13—C10	−1.6 (4)
O14^i^—V1—O10—V5	−6.3 (3)	O15—C10—N13—C9	0.5 (4)
V3^i^—V1—O10—V5	178.39 (3)	N14—C10—N13—C9	−179.9 (3)
O2—V2—O12—V3	−175.73 (8)		

Symmetry code: (i) −*x*, −*y*+1, −*z*+1.

### Hydrogen-bond geometry (Å, º)

**Table:**

*D*—H···*A*	*D*—H	H···*A*	*D*···*A*	*D*—H···*A*
N1—H1*A*···O7	0.89 (3)	1.99 (3)	2.876 (3)	170 (3)
N1—H1*B*···O3^ii^	0.79 (3)	2.40 (3)	3.041 (3)	140 (3)
N2—H2*B*···O12^ii^	0.85 (3)	1.99 (3)	2.835 (3)	176 (3)
N4—H4*A*···O16^iii^	0.87 (3)	2.03 (3)	2.893 (3)	176 (3)
N4—H4*B*···O17	0.79 (3)	2.13 (3)	2.908 (3)	168 (3)
N6—H6*A*···O15^iv^	0.91 (3)	2.08 (3)	2.984 (3)	171 (3)
N6—H6*B*···O9^v^	0.80 (3)	2.15 (3)	2.947 (3)	173 (3)
N7—H7*A*···O5^v^	0.75 (3)	2.51 (3)	3.059 (3)	131 (3)
N7—H7*A*···O10^v^	0.75 (3)	2.64 (3)	3.362 (3)	161 (3)
N7—H7*B*···O13	0.81 (3)	2.23 (3)	3.031 (3)	169 (3)
N9—H9*A*···O17^vi^	0.84 (3)	2.06 (3)	2.886 (3)	170 (3)
N9—H9*B*···O16	0.81 (3)	2.10 (3)	2.889 (3)	165 (3)
N11—H11*A*···O1^vii^	0.84 (3)	2.59 (3)	3.175 (3)	128 (2)
N11—H11*A*···O11^vii^	0.84 (3)	2.05 (3)	2.868 (2)	162 (3)
N11—H11*B*···O1	0.70 (3)	2.48 (3)	3.049 (2)	140 (3)
N11—H11*B*···O10	0.70 (3)	2.45 (3)	3.093 (2)	153 (3)
N12—H12*A*···O15	0.83 (3)	1.93 (3)	2.613 (3)	139 (3)
N12—H12*B*···O4^ii^	0.82 (3)	2.52 (4)	3.231 (3)	145 (3)
N12—H12*B*···O11^vii^	0.82 (3)	2.60 (3)	3.271 (3)	140 (3)
N13—H13···O6	0.82 (3)	2.11 (3)	2.926 (2)	175 (3)
N14—H14*A*···N8^iv^	0.88 (4)	2.22 (4)	3.091 (3)	175 (3)
N14—H14*B*···O2^i^	0.74 (3)	2.44 (4)	3.055 (3)	140 (4)
N14—H14*B*···O4	0.74 (3)	2.53 (3)	3.156 (3)	143 (3)
O16—H16*A*···O8	0.80 (3)	1.97 (3)	2.770 (2)	178 (3)
O16—H16*B*···O3^vi^	0.71 (3)	2.23 (3)	2.929 (2)	171 (4)
O17—H17*A*···O8	0.75 (3)	2.12 (3)	2.861 (2)	170 (3)
O17—H17*B*···O5^iii^	0.78 (3)	2.13 (3)	2.866 (2)	159 (3)

Symmetry codes: (i) −*x*, −*y*+1, −*z*+1; (ii) *x*−1, *y*, *z*; (iii) −*x*, −*y*+2, −*z*+1; (iv) −*x*, −*y*+1, −*z*; (v) *x*+1, *y*, *z*; (vi) −*x*+1, −*y*+2, −*z*+1; (vii) −*x*−1, −*y*+1, −*z*+1.
